# Does Surgery-First Orthognathic Approach Improve Quality of Life of Orthodontic Patients With Skeletal Class III Malocclusion? A Systematic Review Following Preferred Reporting Items for Systematic Reviews and Meta-Analysis (PRISMA) Guidelines

**DOI:** 10.7759/cureus.81433

**Published:** 2025-03-29

**Authors:** Ahmed S Khalil, Rawan S Alrehaili, Mohammed Bajunaid, Mohammed Alhazmi, Abdullah Alshami, Badr Alharthy, Omar Fakhry, Yara Olfat, Abdulkader Taher, Razan Alotaibi, Maryam Alrefai, Abdulaziz A Barashid

**Affiliations:** 1 Orthodontics, Private Practice, Medina, SAU; 2 Dentistry, Private Practice, Medina, SAU; 3 College of Dentistry, King Abdulaziz University, Jeddah, SAU; 4 College of Dentistry, Batterjee Medical College, Jeddah, SAU; 5 College of Dentistry, King Saud Bin Abdulaziz University for Health Sciences, Riyadh, SAU; 6 Dentistry, Ministry of Health, Al Hofuf, SAU; 7 Orthodontics and Dentofacial Orthopedics, King Abdulaziz University, Jeddah, SAU

**Keywords:** class iii malocclusion, mandibular setback, maxillary advancement, orthodontics, orthognathic surgery, psychosocial outcomes, quality of life, surgery-first approach

## Abstract

The surgery-first approach has gained popularity in recent years due to its ability to bypass the presurgical orthodontic phase and significantly reduce treatment duration. However, its broader impact on quality of life and psychosocial outcomes in patients with skeletal class III malocclusion has not been systematically evaluated. This systematic review aimed to assess the effects of the surgery-first approach on quality of life, psychosocial outcomes, and treatment duration in this patient population. The review was conducted in accordance with Preferred Reporting Items for Systematic Reviews and Meta-Analysis (PRISMA) guidelines. A comprehensive search of PubMed, Cochrane Library, Scopus, Web of Science, and Embase databases identified studies evaluating the surgery-first approach in skeletal class III patients. Studies addressing the impact of this approach on quality of life, psychosocial outcomes, and treatment duration were included, and the quality of evidence was assessed using the Newcastle-Ottawa Scale. Due to heterogeneity in study designs and outcomes, a narrative synthesis of the data was performed.

A total of eight studies, conducted between 2015 and 2022, with 252 participants, met the inclusion criteria. The surgery-first approach demonstrated significant improvements in quality of life, particularly in the early postoperative stages, while avoiding the decline typically observed during the presurgical phase of the conventional orthodontics-first approach. Psychosocial benefits, such as reduced anxiety and enhanced self-esteem, were consistently reported. Treatment duration for the surgery-first approach ranged from 7 to 15 months, significantly shorter than the conventional approach. However, the strength of the evidence was limited by small sample sizes and a lack of randomization in most studies.

The surgery-first approach offers distinct advantages for skeletal class III patients, including improved quality of life, enhanced psychosocial outcomes, and shorter treatment duration compared to the conventional orthodontics-first protocol. Nevertheless, further high-quality randomized clinical studies with longer follow-up periods are needed to evaluate long-term outcomes and ensure treatment stability.

## Introduction and background

Skeletal class III malocclusion is a complex dentofacial deformity characterized by a skeletal jaw discrepancy presented as mandibular prognathism, maxillary retrognathism, or a combination of both [1,2]. This jaw discrepancy poses significant functional and aesthetic challenges, impacting facial harmony, self-confidence, and overall quality of life [3,4]. Orthognathic surgery is usually performed to correct such discrepancies, combining orthodontic and surgical interventions to improve occlusion and achieve a more harmonious facial profile [5]. This comprehensive approach is typically reserved for cases where conventional orthodontic treatment alone is insufficient [6-8]. The conventional orthognathic surgery protocol involves a sequence of presurgical orthodontic treatment, surgical correction, and postsurgical orthodontic therapy. Presurgical orthodontics, first described by Worms et al. in 1976, is employed to align and decompensate the dentition prior to surgery [9]. However, while this approach is effective in improving chewing, speech, respiratory functions, and aesthetics [10,11], it is associated with several drawbacks, including lengthy treatment duration and a temporary worsening of facial aesthetics and dental function during the preparatory phase [11-13]. This can negatively impact patients’ psychological well-being and quality of life [14].

To address these drawbacks, the surgery-first orthognathic approach has been introduced as an alternative [15]. This approach eliminates the presurgical orthodontic phase, performing orthognathic surgery first, followed by regular postsurgical orthodontic therapy [16]. By bypassing the lengthy preparatory phase, the surgery-first approach enables immediate improvements in facial aesthetics and function, which can significantly enhance patient satisfaction early in the treatment [17]. Additionally, the biological response triggered by surgery, known as the regional acceleratory phenomenon, accelerates postoperative tooth movement, further reducing the overall treatment duration [18,19]. Nevertheless, it is recommended primarily for carefully selected cases, particularly those with a flat curve of Spee and minimal dental crowding [20].

Quality of life became an essential outcome measure in managing skeletal discrepancies, encompassing an individual’s perception of their physical, emotional, and social well-being within the context of their cultural and personal aspirations [21]. Health-related quality of life is now recognized as a key indicator of treatment success in orthognathic surgery, alongside functional and aesthetic outcomes [22-24]. While the surgery-first approach has gained popularity in recent years due to its ability to reduce treatment duration, its broader impact on quality of life and psychosocial outcomes requires further exploration. Completing treatment within a year while enhancing patient satisfaction is a significant advantage of the surgery-first approach [25]. This surgical protocol has consistently demonstrated improvements in the quality of life for patients undergoing this treatment protocol [26].

Although several reviews have been published on the impact of the surgery-first orthognathic approach on the quality of life, they have generally addressed a range of malocclusions without focusing specifically on patients with skeletal class III malocclusion. Gaining a clear understanding of this approach will enable clinicians to make informed, evidence-based decisions, refine treatment protocols, and more effectively meet the needs of patients with skeletal class III malocclusion indicated for orthognathic surgery. Therefore, the aim of this systematic review was to evaluate the impact of the surgery-first orthognathic approach on the quality of life of orthodontic patients with skeletal class III malocclusion, as well as to examine its associated psychosocial outcomes and treatment duration.

## Review

Methods

Eligibility Criteria

This systematic review was conducted following the Preferred Reporting Items for Systematic Reviews and Meta-Analyses (PRISMA) guidelines [27] and was registered to the Open Science Framework database (osf.io/52ca3). The aim of the current review was to explore the key questions: What impact does the surgery-first orthognathic approach have on the quality of life of orthodontic patients with skeletal class III malocclusion? And what are its associated psychosocial outcomes and treatment duration? Study selection criteria were established using the PICOS framework (Population, Intervention, Comparison, Outcome, Study design) to ensure a structured analysis.

The population of interest included orthodontic patients with skeletal class III malocclusion undergoing orthognathic surgery. The intervention evaluated was the surgery-first orthognathic approach, which is defined as performing orthognathic surgery prior to orthodontic treatment. The comparator was the conventional orthodontics-first approach, where presurgical orthodontic treatment is completed before surgery. The primary outcome assessed was the impact of the surgery-first orthognathic approach on quality of life, measured using validated tools such as the Orthognathic Quality of Life Questionnaire (OQLQ) or similar instruments. Secondary outcomes included psychosocial effects and treatment duration. Eligible study designs included prospective, retrospective, and observational studies.

The review specifically focused on patients with skeletal class III malocclusion in which quality of life was assessed as an outcome using validated tools. Therefore, studies involving patients with syndromes, other malocclusions, or with a lack of data on quality of life were excluded. Additionally, only studies published in English and available in full text were considered, while case reports, case series, editorials, commentaries, and reviews were not included in the analysis.

Information Sources and Search Strategy

A comprehensive electronic search was conducted across multiple databases, including PubMed, Web of Science, Scopus, Embase, and Cochrane Library to identify relevant studies published up to November 30, 2024. The primary objective of the search was to evaluate the impact of the surgery-first orthognathic approach on the quality of life of orthodontic patients with skeletal class III malocclusion. Additionally, it aimed to analyze associated outcomes such as psychosocial effects, treatment duration, and postsurgical stability. Additionally, the reference lists of the included studies were manually screened to identify any supplementary relevant articles. The search strategy in PubMed utilized a combination of keywords and MeSH terms to ensure comprehensive retrieval of studies. The syntax included terms such as: (("surgery-first approach" OR "orthognathic surgery-first" OR "surgical-first protocol") AND ("orthognathic surgery"(MeSH Terms) OR "jaw surgery" OR "maxillofacial surgery") AND ("skeletal class III"(MeSH Terms) OR "class III malocclusion" OR "mandibular prognathism" OR "maxillary retrognathism") AND ("quality of life"(MeSH Terms) OR "QOL" OR "oral health-related quality of life" OR "OHRQoL" OR ") AND ("psychosocial outcomes" OR "treatment outcomes" OR "treatment effects" OR "Orthognathic Quality of Life Questionnaire" OR "OQLQ")). This syntax was adapted for each database to align with its specific indexing system and search requirements.

Study Selection and Assessment

The titles and abstracts of all retrieved studies were evaluated for relevance by two independent researchers. Studies that did not meet the inclusion criteria or were irrelevant to the research question were excluded at this stage. For potentially eligible studies, the full texts were retrieved and assessed independently by the two researchers to determine inclusion based on the predefined eligibility criteria. Disagreements during the screening and selection process were resolved through discussion.

Data Extraction

Data extraction was carried out independently by two researchers to ensure accuracy and reliability. Any discrepancies between the reviewers during the extraction process were resolved through discussion. For each included study, the following key information was systematically recorded: the main author, year of publication, country of origin, study setting, study design, sample size, gender distribution, age, intervention details, comparator group, treatment duration, postsurgical stability, results, and key findings.

Quality Assessment

The methodological quality and risk of bias of the included studies were independently assessed by two researchers using the Newcastle-Ottawa Scale [28], which evaluates studies across three key domains: selection of participants, comparability of study groups, and ascertainment of outcomes. Each study could earn a maximum of nine stars, with four stars allocated to the selection domain, two stars to comparability, and three stars to the outcome domain. Studies scoring 7-9 stars were classified as having a low risk of bias, scores of 4-6 stars indicated a moderate risk of bias, and studies with fewer than 4 stars were considered to have a high risk of bias. Any discrepancies between the reviewers' assessments were discussed and resolved with discussion.

Results

Study Selection

**Figure 1:**
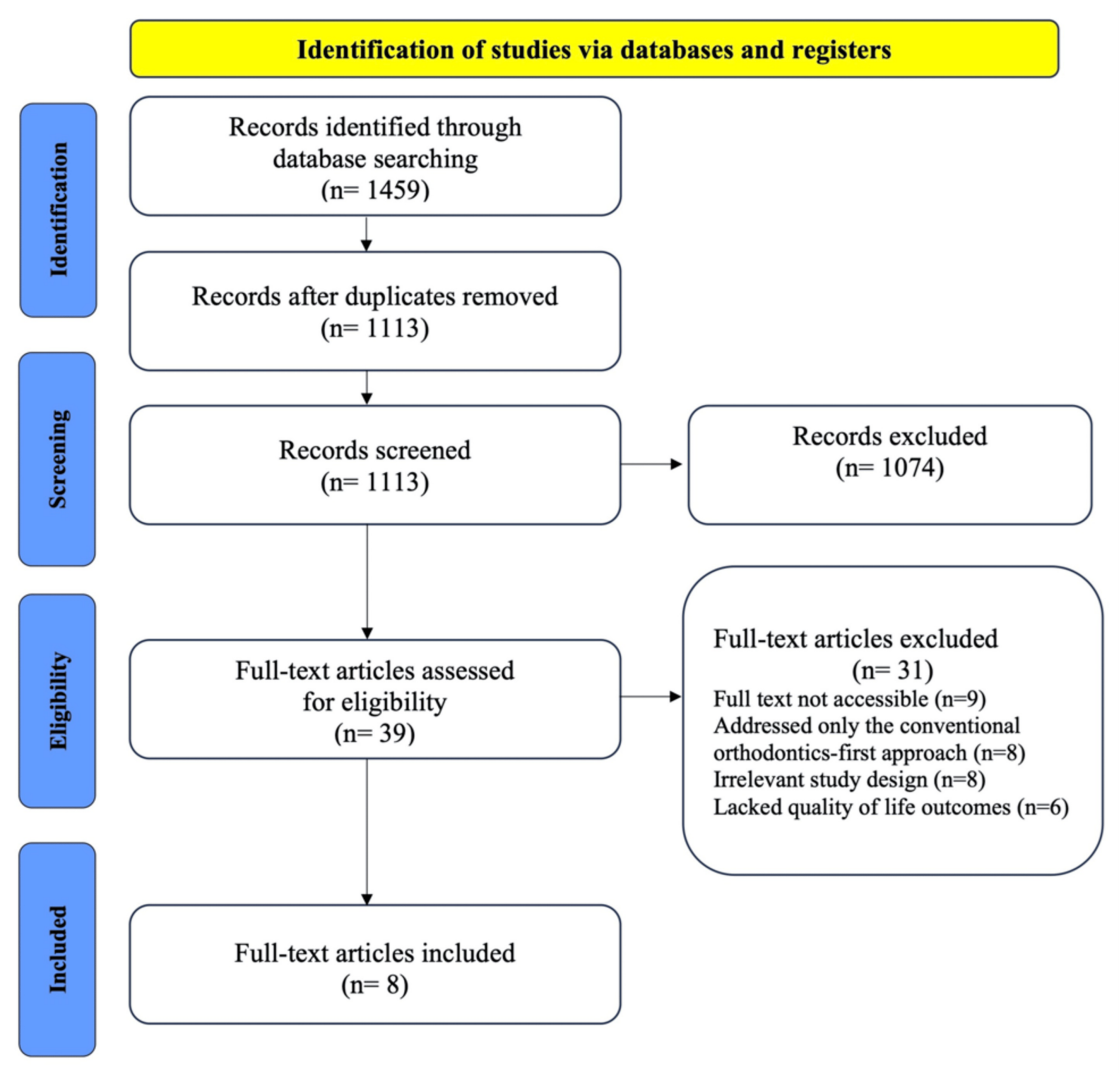
PRISMA flowchart outlining the study selection process PRISMA: Preferred Reporting Items for Systematic Reviews and Meta-Analysis

A total of 1459 records were identified through electronic database searches. After the removal of 346 duplicate records, 1113 records remained for initial screening. Titles and abstracts of these records were evaluated for relevance, resulting in the exclusion of 1074 records that did not meet the inclusion criteria. Following this, 39 full-text articles were retrieved and assessed for eligibility. Of these, 31 articles were excluded for the following reasons: nine articles had inaccessible full texts, eight focused solely on the conventional orthodontics-first approach, eight had irrelevant study design, and six lacked quality-of-life outcomes. Ultimately, eight studies met the inclusion criteria and were included in the systematic review [29-36]. The complete study selection process is depicted in Figure 1.

Study Characteristics

All eight studies included in this systematic review were conducted between 2015 and 2022 across diverse regions including South Korea [29], China [30,35], Brazil [31], Germany [32], Italy [33], United Kingdom [34], and Turkey [36], with a total of 252 participants. All studies assessed the impact of the surgery-first orthognathic approach on the quality of life in patients with skeletal class III malocclusion. The included studies were primarily prospective cohort studies, with the exception of two that were retrospective cohort studies [29,35]. The studies were predominantly conducted in university settings, except for one study conducted in a multidisciplinary orthognathic clinic [34] (Table 1).

**Table 1:** Demographic data of the included studies

Author and Year	Location	Study Setting	Study Design
Park et al., 2015 [29]	South Korea	University setting - School of Dentistry, Seoul National University	Retrospective cohort study
Huang et al., 2016 [30]	China	University setting - Department of Orthodontics, Stomatology Hospital, Wenzhou Medical University	Prospective cohort study
Feu et al., 2017 [31]	Brazil	University setting - Vila Velha University, State University of Rio de Janeiro, Federal University of Rio Grande do Sul	Prospective cohort study
Zingler et al., 2017 [32]	Germany	University setting - Department of Orthodontics and Oral and Maxillofacial Surgery, University Hospital Heidelberg	Prospective cohort study
Brucoli et al., 2019 [33]	Italy	University setting - Division of Oral and Maxillofacial Surgery, University of Eastern Piedmont	Prospective cohort study
Saghafi et al., 2020 [34]	United Kingdom	Multidisciplinary orthognathic clinic	Prospective cohort study
Hu et al., 2021 [35]	China	University setting - Affiliated Hospital of Stomatology, Nanjing Medical University	Retrospective cohort study
Findik et al., 2022 [36]	Turkey	University setting - Suleyman Demirel University and Antalya Bilim University	Prospective cohort study

All studies evaluated the surgery-first orthognathic approach, which involves performing orthognathic surgery prior to orthodontic treatment. This was compared in all included studies to the conventional orthodontics-first approach, where preoperative orthodontics was used for alignment and decompensation prior to surgery with the exception of one study [32]. The included studies had sample sizes ranging from 9 participants [32] to 54 participants [35]. The majority of studies indicated a slight predominance of female participants. However, one study did not provide information on gender distribution [34]. Participants’ ages ranged from 17 to 47 years, with most studies reporting a mean age in the early 20s.

Various surgical techniques were utilized, including Le Fort I osteotomy, bilateral sagittal split osteotomy, and genioplasty. The primary outcome in all studies was quality of life, assessed using validated tools such as the OQLQ, Oral Health Impact Profile (OHIP-14), and SF-36 Health Survey. The duration of treatment varied between the two surgical approaches. For the surgery-first approach, treatment duration ranged from 7 months [36] to 15 months [32], whereas for the conventional orthodontics-first approach the duration ranged from 13 months [29] to 27 months [35]. Follow-up period across the studies ranged from 6 months to 2 years and most of the included studies did not report data on relapse rate (Tables 2-3).

**Table 2:** Characteristics of the included studies SFA: surgery-first approach; COFA: Conventional orthodontics-first approach; NR: not reported

Main Author and Year	Participants (Sample Size and Gender Distribution)	Age (Mean ± SD, Years)	Intervention	Control	Outcome
Park et al., 2015 [29]	N=26 (11 in the SFA: 2 males, 9 females; 15 in the COFA: 3 males, 12 females)	SFA: 26.27 ± 4.45 COFA: 25.00 ± 3.25	Two-jaw surgery: Le Fort I osteotomy, bilateral sagittal split ramus osteotomy	Conventional orthodontics-first approach	Orthognathic Quality of Life Questionnaire (OQLQ)
Huang et al., 2016 [30]	N=50 (25 in the SFA: 13 males, 12 females; 25 in the COFA: 12 males, 13 females)	SFA: 24.2 ± 5.8 COFA: 25.2 ± 4.2	Bilateral sagittal split ramus osteotomy	Conventional orthodontics-first approach	OQLQ, Dental Impact on Daily Living (DIDL)
Feu et al., 2017 [31]	N=16 (8 in the SFA: 4 males, 4 females; 8 in the COFA: 4 males, 4 females)	SFA: 22.9 ± 5.4 COFA: 26.8 ± 7.1	Two-jaw surgery: maxillary advancement and mandibular setback	Conventional orthodontics-first approach	OQLQ, Oral Health Impact Profile (OHIP-14)
Zingler et al., 2017 [32]	N=9 (6 females, 3 males)	26.7 ± 8.4	Le Fort I osteotomy, high oblique sagittal split osteotomy, genioplasty	None	OQLQ, Sense of Coherence (SOC-29), self-perception survey
Brucoli et al., 2019 [33]	N=33 (8 in the SFA: 4 males, 4 females; 25 in the COFA: 6 males, 19 females)	SFA: 35.63 ± 13.45 COFA: 25.04 ± 5.58	NR	Conventional orthodontics-first approach	Short Form Health Survey (SF-36), Beck Depression Inventory-II (BDI-II), Resilience Scale for Adults (RSA), Oral Health Impact Profile (OHIP-14)
Saghafi et al., 2020 [34]	N=32 (18 in the SFA, 14 in the COFA) Exact gender distribution NR	17–47 (specific mean with SD NR)	Le Fort I osteotomy, bilateral sagittal split ramus osteotomy, genioplasty	Conventional orthodontics-first approach	OQLQ, Generalized Anxiety Disorder-7 (GAD-7), Patient Health Questionnaire-9 (PHQ-9)
Hu et al., 2021 [35]	N=54 (28 in the SFA: 12 males, 16 females; 26 in the COFA: 12 males, 14 females)	SFA: 23.8, COFA: 21.9	Bimaxillary orthognathic surgery: Le Fort I osteotomy, bilateral sagittal split osteotomy, genioplasty	Conventional orthodontics-first approach	OQLQ, cost-effectiveness analysis
Findik et al., 2022 [36]	N=32 (14 in the SFA group: 9 males, 5 females; 18 in the COFA: 9 males, 9 females)	SFA: 23.04 ± 3.36 COFA: 29.27 ± 3.78	Two-jaw surgery: Le Fort I osteotomy, bilateral sagittal split ramus osteotomy	Conventional orthodontics-first approach	OQLQ, Psychosocial Impact of Dental Aesthetics Questionnaire (PIDAQ), Oral Health Impact Profile (OHIP-14), Beck Depression Inventory-II (BDI-II), Rosenberg Self-Esteem Scale (RSES)

**Table 3:** Secondary outcomes and results reported in the included studies SFA: surgery-first approach; COFA: Conventional orthodontics-first approach; NR: not reported

Author and Year	Average Treatment Duration (Months)	Orthodontic Treatment Performed Prior to Surgery	Relapse Rate	Results
Park et al., 2015 [29]	SFA: 10 COFA: 13	SFA: orthodontics initiated postoperatively COFA: Preoperative orthodontics for alignment and decompensation	NR	SFA showed no deterioration in quality of life during the preoperative phase and improved outcomes postoperatively compared to COFA.
Huang et al., 2016 [30]	SFA: 12 COFA: 24	SFA: Orthodontic brackets placed immediately before surgery COFA: Preoperative orthodontics for alignment and decompensation	NR	Both approaches significantly improved quality of life, but SFA avoided deterioration in quality-of-life pre-surgery.
Feu et al., 2017 [31]	SFA: 12 COFA: NR	SFA: Orthodontic brackets placed immediately before surgery; postoperative orthodontics initiated 2 weeks after surgery. COFA: Preoperative orthodontics for alignment and decompensation	NR	Significant improvements in oral health-related quality of life observed in the SFA group, with progressive improvements throughout follow-up.
Zingler et al., 2017 [32]	15.7 ± 3.3	Orthodontic brackets placed one week prior to surgery with an inactive stainless steel archwire. Postoperative orthodontics commenced early	No significant relapse observed	Significant improvement in quality of life and sense of coherence.
Brucoli et al., 2019 [33]	SFA: 12 COFA: 24	SFA: Immediate postoperative orthodontics after surgery COFA: Preoperative orthodontics for alignment and decompensation	NR	SFA resulted in faster improvements in quality of life, reduced depressive symptoms, and better psychological outcomes compared to COFA.
Saghafi et al., 2020 [34]	NR	SFA: Orthodontic brackets applied immediately before surgery COFA: Preoperative orthodontics for alignment and decompensation	NR	SFA eliminated social deterioration seen in the presurgical phase of COFA and showed significant improvements in quality-of-life post-surgery.
Hu et al., 2021 [35]	SFA: 12.47 COFA: 27.67	SFA: Orthodontic brackets applied immediately before surgery COFA: Preoperative alignment and decompensation	No significant skeletal or dental relapse observed	SFA showed faster quality-of-life improvements and reduced overall treatment time and cost compared to COFA.
Findik et al., 2022 [36]	SFA: 7 COFA: 20	SFA: Orthodontic brackets placed 1 day before surgery; postoperative orthodontics started 15 days after surgery COFA: Preoperative orthodontics for alignment and decompensation	NR	The SFA group experienced faster psychosocial and quality-of-life improvements compared to COFA, attributed to reduced treatment time.

Quality Assessment

Overall, five studies were determined to have a low risk of bias with a score of 7 stars [30,31,33,35,36]. The remaining three studies were categorized as having a moderate risk of bias, scoring 6 stars as depicted in Table 4 [29,32,34]. Most studies demonstrated strong methodology in the selection domain, with three studies achieving the maximum score of 4 stars [30,33,36]. The remaining five studies scored 3 stars due to limitations in the representativeness of the cohort or issues with the ascertainment of exposure [29,31,32,34,35]. In terms of comparability, all studies received 1 star, as they controlled for at least one key confounder, such as age, gender, or baseline malocclusion severity. However, none of the studies achieved the maximum score of 2 stars. For the outcome domain, three studies scored the maximum 3 stars, demonstrating robust outcome assessment and adequate follow-up periods [31,32,35]. The remaining studies scored 2 stars, primarily due to shorter follow-up durations or limitations in follow-up [29,30,33,34,36].

**Table 4:** Risk of bias assessments of the studies using the Newcastle-Ottawa Scale ★ indicates the star rating based on the Newcastle-Ottawa Scale

Author and Year	Selection (4★)	Comparability (2★)	Outcome (3★)	Total Score (9★)	Risk of Bias
Park et al., 2015 [29]	★★★	★	★★	6	Moderate risk
Huang et al., 2016 [30]	★★★★	★	★★	7	Low risk
Feu et al., 2017 [31]	★★★	★	★★★	7	Low risk
Zingler et al., 2017 [32]	★★★		★★★	6	Moderate risk
Brucoli et al., 2019 [33]	★★★★	★	★★	7	Low risk
Saghafi et al., 2020 [34]	★★★	★	★★	6	Moderate risk
Hu et al., 2021 [35]	★★★	★	★★★	7	Low risk
Findik et al., 2022 [36]	★★★★	★	★★	7	Low risk

Results of Individual Studies and Main Findings

A quantitative meta-analysis was not performed due to substantial heterogeneity across the included studies. Variations in study designs, treatment protocols, patient demographics, and reported outcomes contributed to the lack of uniformity, making statistical pooling of results unfeasible. Park et al. demonstrated that the surgery-first approach improved quality of life postoperatively while preventing the presurgical decline in quality of life typically observed in the conventional orthodontics-first approach [29]. In contrast, the conventional orthodontics-first approach showed temporary deterioration in quality of life during the presurgical orthodontic phase but achieved similar improvements postoperatively. Huang et al. also highlighted the lack of diminish of presurgical quality of life with the surgery-first approach, whereas the conventional orthodontics-first approach group experienced a marked reduction in quality of life before surgery despite comparable postoperative improvements [30]. Feu et al. emphasized that the surgery-first approach led to progressive and significant enhancements in oral health-related quality of life, while conventional orthodontics-first approach patients experienced worsened malocclusion during the preoperative orthodontic preparation period [31]. Similarly, Zingler et al. found that the surgery-first approach resulted in significant improvements in quality of life within three months postoperatively, with additional benefits in social coherence and oral function [32]. These changes were attributed to accelerated tooth movement facilitated by bone remodeling. Brucoli et al. reported that surgery-first approach patients achieved faster psychological recovery and quality-of-life improvements compared to conventional orthodontics-first approach patients, who required a longer treatment duration to reach similar outcomes [33]. Saghafi et al. corroborated these findings, noting that the surgery-first approach significantly enhanced quality-of-life postoperatively and mitigated the decline in social quality of life commonly observed in the conventional orthodontics-first approach before surgery [34].

Hu et al. demonstrated that both approaches improved quality of life post-treatment, but the surgery-first approach was more cost-effective and associated with a shorter treatment duration [35]. Finally, Findik et al. found that the surgery-first approach resulted in faster psychosocial recovery and quality-of-life improvements than the conventional orthodontics-first approach, largely due to its shorter treatment time [36]. Table 5 provides a summary of the key findings from the included studies.

**Table 5:** Key findings of the included studies

Author and Year	Main Findings
Park et al., 2015 [29]	The surgery-first approach improved the quality of life postoperatively and prevented its decline before surgery, unlike the conventional three-stage method, which showed temporary presurgical deterioration but similar postoperative improvements.
Huang et al., 2016 [30]	Quality of life improved significantly postoperatively in both groups, but the surgery-first approach avoided the presurgical quality of life decline seen in the orthodontics-first approach.
Feu et al., 2017 [31]	The surgery-first approach led to progressive and significant improvements in oral health-related quality of life, while patients in the orthodontics-first approach group experienced stagnation in quality of life and worsened malocclusion during preoperative preparation.
Zingler et al., 2017 [32]	Quality of life improved significantly within three months after surgery with the surgery-first approach, along with enhanced social coherence and oral function. Accelerated tooth movement was observed due to bone remodeling factors.
Brucoli et al., 2019 [33]	Patients in the surgery-first approach group experienced faster improvements in quality of life and psychological recovery compared to those in the conventional treatment method group, which required a longer treatment duration.
Saghafi et al., 2020 [34]	The surgery-first approach significantly improved the quality of life postoperatively and prevented the social quality of life decline observed in the orthodontics-first approach before surgery.
Hu et al., 2021 [35]	Both approaches improved the quality of life post-treatment, but the surgery-first approach was more cost-effective and had a shorter treatment duration compared to the orthodontics-first approach.
Findik et al., 2022 [36]	The surgery-first approach resulted in faster improvements in psychosocial outcomes and quality of life compared to the conventional orthognathic surgery method, attributed to shorter treatment time.

Discussion

Most of the studies included in this systematic review, as well as some for which full texts were unavailable, were published within the past decade. This indicated a growing interest in the surgery-first orthognathic approach. Although several systematic reviews have been conducted on this topic, most have predominantly concentrated on treatment-related aspects, such as duration, rather than highlighting the quality of life and psychological effects associated with this approach. Furthermore, previous reviews often included studies on various malocclusions without specifically addressing skeletal class III patients. Consequently, this systematic review aimed to fill this gap by providing a more focused, contemporary, and up-to-date analysis of the impact of the surgery-first approach on the quality of life of orthodontic patients with skeletal class III malocclusion. Patients treated with the surgery-first approach showed significant improvements in quality of life, particularly in facial aesthetics, oral function, and social confidence in all studies. The findings of the included studies highlighted significant differences in the patient experience of surgery-first and orthodontics-first approaches, particularly concerning quality of life and compliance challenges. The orthodontics-first approach was frequently associated with a deterioration in quality of life during the presurgical orthodontic phase [37-39]. This decline was primarily attributed to the necessary decompensation of the dentition, which often worsens the facial profile and exacerbates patients' dissatisfaction with their appearance [40,41]. One should emphasize that this phase not only delays the visible benefits of treatment but also imposes a psychological burden, as patients endure an extended period of compromised aesthetics and oral function. Conversely, the surgery-first approach addresses these challenges by prioritizing immediate correction of skeletal discrepancies and facial aesthetics. All the included studies reported an average treatment duration of up to one year for the surgery-first approach, with the exception of the study by Zingler et al [32]. Postsurgical orthodontics capitalizes on the regional acceleratory phenomenon which facilitates faster tooth movement due to enhanced bone remodeling processes initiated by surgical trauma in the operated areas and consequently reduces total treatment duration [42-44]. Interleukin-1 and interleukin-6, which are associated with early inflammation following bone injury, were observed to increase in crevicular fluids directly after surgery [32].

It is worth mentioning that ideal candidates for the surgery-first approach are those with mild to moderate dental crowding, minimal vertical or transverse asymmetries, and a flat curve of Spee [45,46]. This ensures that the skeletal correction achieved during surgery can be effectively complemented by subsequent orthodontic alignment. Conversely, cases involving severe asymmetries or complex malocclusions may be better suited to the orthodontics-first approach to achieve optimal functional outcomes [47,48]. However, the surgery-first approach also presents unique challenges, especially with patient compliance during postsurgical orthodontic treatment. After surgery, patients may perceive their treatment as largely complete due to the immediate improvement in appearance and function. This perception can lead to a lack of motivation to adhere to the postsurgical orthodontic phase, potentially compromising long-term outcomes. This issue aligns with the findings of Kayak et al. [49], who noted that around nine months after orthognathic surgery following the orthodontics-first protocol, orthodontists may start encountering challenges with patient compliance. Moreover, the residual malocclusion often left untreated due to the absence of presurgical decompensation requires meticulous and precise orthodontic management post-surgery, which some patients may find demanding [31]. In contrast, Jeyaraj and Chakranarayan observed that patients, inspired by the significant aesthetic improvements achieved after surgery, often exhibit strong dedication and compliance with postsurgical orthodontic treatment, even when it is prolonged [50]. It is important to acknowledge the significance of selecting treatment protocols based on individual patient needs, ensuring a balance between clinical outcomes and psychosocial considerations. While the surgery-first approach offers substantial benefits - particularly in improving quality of life and reducing treatment duration - its success is contingent upon careful case selection and the effective management of the postsurgical orthodontic phase.

Limitations

The evidence included in the current review is subject to several limitations. None of the studies employed randomization, increasing the risk of selection bias and limiting the ability to establish causality. The retrospective designs used in some studies [29,35] might introduce selection bias by potentially favoring patients with complete and successful treatment records. Additionally, the sample size of the included studies was relatively small, which may limit the generalizability of the findings. Variability in surgical procedures with one-jaw surgery or two-jaw surgery, outcome measures, and follow-up durations across studies contributed to the heterogeneity. While questionnaires are essential tools for assessing patient-reported their inherent subjectivity, variability in application, and potential lack of standardization across studies represent limitations in the evidence base. Most studies did not objectively assess the quality of orthodontic outcomes, which might have affected the overall results. Furthermore, most studies did not account for potential confounding factors, such as patient compliance, psychosocial background, or socioeconomic influences, which could impact the outcomes.

While this review followed rigorous methods by including comprehensive database searches and standardized quality assessment using the Newcastle-Ottawa Scale, there are inherent limitations. The restriction to English-language studies may have excluded relevant evidence published in other languages. The decision to forgo a meta-analysis due to heterogeneity limited the quantitative synthesis of results and prevented the pooling of effect size.

Implications of the results for practice and future research

The findings of this review highlight the benefits of the surgery-first orthognathic approach, including expedited improvements in quality of life and reduced treatment duration compared to the conventional orthodontics-first approach. For clinical practice, these findings suggest that the surgery-first approach should be considered for patients seeking quicker functional and psychosocial recovery, provided they meet the criteria for this protocol. Clinicians could advocate for further training and resources to implement this approach more widely. However, careful case selection remains crucial as this approach is not suitable for all patients. Larger multi-center randomized controlled trials are necessary to confirm the findings. Future studies should adopt standardized treatment protocols and validated outcome measures to facilitate comparability. Extended follow-up periods are also essential to assess long-term outcomes, such as stability and sustained quality of life improvements. Research should focus on integrating standardized measures to evaluate the quality of orthodontic outcomes and their correlation with patient-reported benefits. Additionally, research should explore patient-specific factors, including compliance and psychosocial influences, to identify subgroups that may benefit most from the surgery-first approach.

## Conclusions

The results of this systematic review indicate that the surgery-first approach for skeletal class III patients provides notable advantages over the conventional orthodontics-first approach in terms of expedited quality-of-life improvement, enhanced psychosocial outcomes, and reduced treatment duration. By addressing skeletal discrepancies early, this approach also mitigates the presurgical decline in quality of life commonly associated with the conventional orthodontics-first method. However, both approaches yielded comparable long-term postoperative quality-of-life improvements. The current body of evidence is limited by a lack of randomization, small sample sizes, and inconsistencies in study designs. Future research should focus on overcoming these limitations by utilizing well-designed clinical studies, larger study populations, and standardized treatment protocols.
